# Early longitudinal changes in left ventricular function and morphology in diabetic pigs: evaluation by 3.0T magnetic resonance imaging

**DOI:** 10.1186/s12933-022-01734-y

**Published:** 2023-01-10

**Authors:** Wei-Feng Yan, Hua-Yan Xu, Li Jiang, Lu Zhang, Ying-Kun Guo, Yuan Li, Li-Ting Shen, Chen-Yan Min, Zhi-Gang Yang

**Affiliations:** 1 Department of Radiology, West China Hospital, Sichuan University, 37# Guo Xue Xiang, Chengdu, Sichuan 610041 China; 2 Department of Radiology, Key Laboratory of Birth Defects and Related Diseases of Women and Children of Ministry of Education, West China Second University Hospital, Sichuan University, Chengdu, China

**Keywords:** Diabetic cardiomyopathy, Cardiac magnetic resonance, Time‒volume curve, Feature tracking technique, Diabetic pig

## Abstract

**Background:**

Previous researches on large animal models of diabetic cardiomyopathy were insufficient. The aim of this study was to evaluate early changes in left ventricular (LV) function and morphology in diabetic pigs using a cardiac magnetic resonance (CMR) time-volume curve and feature tracking technique.

**Methods:**

Streptozotocin (STZ) was used to induce diabetic in sixteen pigs. 3.0T MRI scanned the pig’s heart before and 2, 6, 10 and 16 months after modelling. CMR biomarkers, including time-volume curve and myocardial strain, were compared to analyse the longitudinal changes in LV function and morphology. Pearson correlation was used to evaluate the relationship between LV strain and remodelling. Cardiac specimens were obtained at 6, 10, and 16 months after modelling to observe the myocardial ultrastructural and microstructure at different courses of diabetes.

**Results:**

Twelve pigs developed diabetes. The 80% diastolic volume recovery rate (DVR) at 6 months after modelling was significantly higher than that before modelling (0.78 ± 0.08vs. 0.67 ± 0.15). The LV global longitudinal peak strain (GLPS) (− 10.21 ± 3.15 vs. − 9.74 ± 2.78 vs. − 9.38 ± 3.71 vs. − 8.71 ± 2.68 vs. − 6.59 ± 2.90%) altered gradually from the baseline data to 2, 6, 10 and 16 months after modelling. After 16 months of modelling, the LV remodelling index (LVRI) of pigs increased compared with that before modelling (2.19 ± 0.97 vs. 1.36 ± 0.45 g/ml). The LVRI and myocardial peak strain were correlated in diabetic pigs (r= − 0.40 to − 0.54), with GLPS being the most significant. Electron microscopy and Masson staining showed that myocardial damage and fibrosis gradually increased with the progression of the disease.

**Conclusion:**

Intravenous injection of STZ can induce a porcine diabetic cardiomyopathy model, mainly characterized by decreased LV diastolic function and strain changes accompanied by myocardial remodelling. The changes in CMR biomarkers could reflect the early myocardial injury of diabetic cardiomyopathy.

**Supplementary Information:**

The online version contains supplementary material available at 10.1186/s12933-022-01734-y.

## Background

Due to the ageing population, changes in lifestyle and the increase in obesity, diabetes is on the rise worldwide [1, 2]. In addition, diabetes easily causes complications in the heart, brain, kidney and other organs. Previous studies have shown that heart failure is the leading cause of poor prognosis and death in patients with diabetes, which makes early diagnosis and evaluation of diabetic cardiomyopathy crucial [3, 4].

Cardiac magnetic resonance (CMR), which is considered the gold standard for evaluating the function and structure of the heart, provides accurate and reproducible measures of cardiac blood flow and morphology characteristics. The time‒volume curve and feature tracking acquired from conventional CMR cine imaging are noninvasive methods for evaluating cardiac systolic and diastolic function, and have been widely used in scientific research [5–7].

Since diabetic patients are often diagnosed with other metabolic diseases, and the interaction of the various factors of those diseases is complex, the mechanism for how hyperglycaemia affects the heart is unclear [8, 9]. To accurately study the pathogenesis and evolution of diabetic cardiomyopathy, it is necessary to establish a suitable animal experimental model. In the past, streptozotocin (STZ)-induced diabetes has been used in many animals. Among them, diabetic pigs are more similar to humans than rodents and are considered an ideal research model [10, 11]. However, as far as we know, there are few CMR longitudinal studies targeting heart injury in diabetic models of pigs.

Therefore, this study attempts to investigate the early dynamic changes in left ventricular (LV) function and morphology in STZ-induced diabetic pigs using the CMR time‒volume curve and feature tracking technique.

## Materials and methods

### Animals and study design

Sixteen 16-week-old female Bama Mini‒pigs with a mean initial body weight of 18.6 ± 5.2 kg were used in this study. Animals were raised in independent polypropylene cages under controlled conditions for experiments. All animal experiments complied with the ARRRIVE guidelines and have been approved by our hospital’s Ethics Committee on Biomedical Research.

This study was a self-controlled study in diabetic pigs. Before modelling, all pigs underwent CMR scanning to obtain baseline data. Then CMR follow-up scans were performed on pigs with diabetes at 2, 6, 10 and 16 months after modelling. Cardiac specimens were obtained from 3 pigs at 6, 10, and 16 months after modelling to observe the cardiac microstructure at different stages of diabetes mellitus.

### Diabetes induction by intravenous injection of STZ

After fasting for 16 h, pigs were anaesthetized by subcutaneous injection of Zoletil 50 (10 mg/kg). An appropriate amount of STZ solution (150 mg/kg) was injected into the ear vein of the pigs at a uniform speed for 5 min. STZ was dissolved in 0.1 mol/l citric acid solution at pH 4.4–4.5. The STZ solution was configured in a dark room and the syringe was wrapped in tin foil during the injection process to ensure drug properties. After injection of STZ, the blood glucose of the auricular vein was monitored by a blood glucose metre at 1 h (h), 2 h, 6 h, 8 h, 12 h, 16 h, 20 h, 24 h, 36 and 48 h. When the blood glucose was lower than 2 mmol/L, 5% glucose 10–20 ml was injected intravenously. All of the pigs began to eat 6 h after STZ injection.

Forty-eight hours after STZ injection, fasting blood glucose (FBG) was tested every morning. To ensure the survival of the animals, 12 U insulin was injected subcutaneously when the FBG was higher than 20 mmol/l, and 20 U was injected when it was higher than 25 mmol/l. One month after the injection of STZ, if a pig’s blood glucose concentration was continuously higher than 7 mmol/l, then the diabetes model was considered to be successful. One week after the first injection of STZ, if the blood glucose of any pig was less than 7 mmol/l, then 100 mg/kg STZ was injected again in the same way. If the FBG of any pig was still not up to 7 mmol/l after two repeated injections of STZ, then it was concluded that the diabetes model failed. Pigs that failed to model diabetes were not included in the subsequent CMR examinations.

### Preparation for the MRI scan

Before CMR examination, pigs were anaesthetized by intramuscular injection of Zoletil 50 (10–15 mg/kg) with atropine (0.3–0.5 mg). Blood samples were collected from the superior vena cava with an aseptic syringe, and FBG, glycosylated haemoglobin (HbAlc), and liver and kidney function were measured.

Endotracheal intubation was performed using a 4.5-6.0 mm endotracheal tube connected to a special animal ventilator for mechanical ventilation. Anaesthesia was sustained by isoflurane inhalation (1.0–2.0%), with a respiratory rate of 10–30 beats/min and an inhalation/breathing ratio of 1:2.

### CMR protocol

CMR imaging of pigs was performed using a 3.0-T whole-body MR system equipped with a commercial 18-channel receiver coil (Magnetom Skyra, Siemens Medical Solutions, Erlangen, Germany). ECG and respiratory gating were connected during image acquisition. Data were acquired during end-inspiratory breath holding. After scout images, a steady-state free precession sequence (echo time, 1.36 ms; repetition time, 3.15 ms; flip angle, 35°; slice thickness, 6.5 mm; matrix, 154 × 192 pixels; and field of view, 400 × 320 mm^2^) with retrospective ECG-gating was used to acquire dynamic cine imaging of the LV for functional analysis. The protocol comprised cine imaging in short axis, 2-, 3-, and 4- chamber views. The LV was entirely imaged from the base to the apex in 9–12 short-axis cine images with 6–8 mm thick contiguous slices.

### Image analysis

An experienced radiologist analysed the CMR data on an offline workstation. All image postprocessing operations were performed following the latest International Cardiac Magnetic Resonance Association guidelines [12]. The images were analysed using offline commercial software (cvi42, v.5.10.2; Circle cardiovascular imaging, Calgary, Canada). The end-systolic and end-diastolic endocardium and epicardium on the short axis were drawn to obtain routine cardiac function indices, including LV EDV, end-systolic volume (ESV), stroke volume (SV), ejection fraction (EF), and LV mass. The LV remodelling index was determined by dividing the LV mass by the LV EDV [13]. At the end of diastole and end systole, the maximum LV diameter was measured from the endocardium of the free wall to the interventricular septum on the four-chamber view. The LV wall thickness was measured at the end of diastole in the interventricular septum of four-chamber view. The time‒volume curve parameters, including the peak ejection rate (PER) and peak filling rate (PFR), were obtained by drawing the endocardial boundary of the LV on each short-axis image. DVR, the proportion of diastole required for recovery of a given percentage (i.e., 80%) of stroke volume, was calculated by importing volume data into Origin software (Origin 8.0, Microcal Software Inc., Northampton, MA, USA) [14]. In addition, the end-diastolic endocardium and epicardium of the short axis and two long-axis sections were drawn to analyse the LV strain parameters, including LV radial global peak strain (GRPS), circumferential global peak strain (GCPS), longitudinal global peak strain (GLPS) and the peak strain rates in those three directions during systole (PSSR) and diastole (PDSR). The interface of LV feature tracking postprocessing is shown in Additional file 1: Fig. S1.

### Histological analysis

At 6, 10 and 16 months after successful modelling, three pigs were randomly selected and sacrificed by intravenous injection of 20 ml potassium chloride under deep anesthesia. After cardiac arrest, the pig’s heart was removed from the chest. Approximately 50 mg of LV apical tissue was placed in glutaraldehyde fixative for 24 h for electron microscopy. To fix the myocardium, the rest of the heart tissue was immersed in 10% formalin solution; then, it was dehydrated and embedded, and 5 mm slices were cut parallel from the apex to the bottom of the heart and made into sections for hematoxylin–eosin (HE) and Masson staining to observe the changes in myocardial histomorphology and tissue composition. The collagen volume fraction (CVF) was calculated by the ImageJ software (U.S. National Institutes of Health).

### Intraobserver and interobserver reproducibility

Two investigators assessed the reproducibility of CMR parameters. To determine the internal variability of the observer, the original images of 15 CMR scans were randomly selected, and the parameters of LV global strain and time‒volume curve were reanalyzed by the same radiologist (LJ) after an interval of 1 month. To determine the variability between observers, another investigator (WFY) reanalyzed the results of the 15 scans. In the variability assessment, each observer was blinded to the pigs’ state and other observers’ findings.

### Statistical analysis

Statistical analyses were performed with IBM SPSS (version 22.0, IBM SPSS Inc., Armonk, New York, USA). All continuous variables were checked for normality using the Kolmogorov‒Smirnov test. Continuous variables are expressed as the mean ± standard deviation. The baseline characteristics and CMR parameters of pigs before and after modelling were compared by one-way analysis of variance (one-way ANOVA) and the Kruskal‒Wallis test. One-way ANOVA was used when the data conformed to the homogeneity of variance and normal distribution assumptions and was followed by the Tukey test. Kruskal‒Wallis tests were used when the data exhibited skewed distributions. Pearson correlation was used to analyse the relationship between LVRI and LV myocardial strain. Inter- and intraobserver agreements were determined by evaluating intraclass correlation coefficients (ICCs). A two-tailed *P* value < 0.05 indicated statistical significance for all tests.

## Results

### Modelling of diabetic pigs

**Fig. 1:**
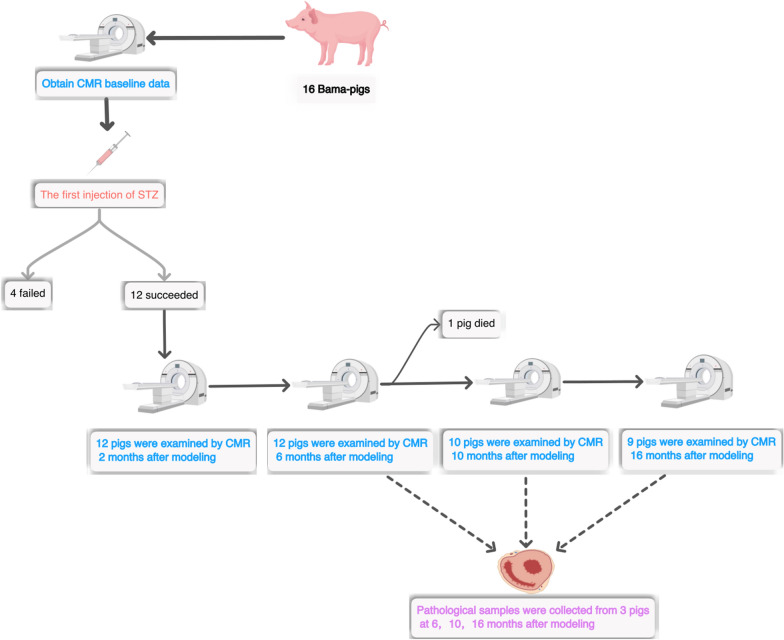
Flow chart of the experimental procedure. After completing the first CMR scan, sixteen pigs were injected with STZ to establish a diabetic model. Twelve diabetic pigs were included in the CMR follow-up study. One pig was randomly selected for heart sampling in each of the last three scans

As shown in Fig. 1, of the sixteen pigs, twelve developed diabetes after the STZ induction. The FBG of the other fourpigs was still less than 7 mmol/l after three injections of STZ, so they were regarded as a failed model and were no longer included in the subsequent follow-up study. A total of 12 diabetic pigs were enrolled in the follow-up study, of which one pig died accidentally 7 months after modelling. The blood glucose changes in each pig during the modelling process are shown in Additional file 1: Fig. S2.

With the progression of the disease, the physiological and biochemical indices of pigs at each CMR scan time are shown in Table 1. The fasting blood glucose of diabetic pigs remained at the level of diabetes. The total serum protein and urea of diabetic pigs after modelling were higher than those before modelling. At 6 months after modelling, the creatine kinase isozymes of diabetic pigs increased.

**Table 1:** Changes in weight and serological indices in pigs with different courses of diabetes

	Before modelling (n = 16)	2 months after modelling (n = 12)	6 months after modelling (n = 12)	10 months after modelling (n = 10)	16 months after modelling (n = 9)	*P* value
Weight (kg)	22.78 ± 5.77	31.82 ± 6.82*	35.92 ± 10.48*	43.65 ± 12.85^*#^	43.17 ± 13.22^*#^	0.001
FBG (mmol/l)	3.72 ± 0.98	12.54 ± 3.03*	13.02 ± 5.03*	16.53 ± 4.89*	16.26 ± 4.21*	0.001
HbA1c (%)	3.01 ± 0.36	3.37 ± 1.02*	5.40 ± 1.37*	4.92 ± 1.37*	5.78 ± 1.15^*#^	0.001
TC (mmol/l)	5.06 ± 5.19	2.62 ± 1.25	3.44 ± 3.31	3.67 ± 4.51	3.30 ± 4.04	0.601
TG (mmol/l)	0.56 ± 0.32	0.94 ± 1.08	1.11 ± 1.58	0.85 ± 1.08	0.67 ± 0.87	0.681
HDL (mmol/l)	1.11 ± 0.57	0.65 ± 0.36	0.53 ± 0.38	1.02 ± 1.71	0.54 ± 0.23	0.215
LDL (mmol/l)	2.45 ± 2.51	5.69 ± 7.67*	1.81 ± 1.81^#^	1.83 ± 1.73^#^	2.42 ± 3.81	0.155
ADA (U/l)	7.90 ± 4.79	4.88 ± 1.80*	2.86 ± 1.00*	2.92 ± 1.26*	3.55 ± 2.09*	0.006
ALT (U/l)	46.44 ± 20.46	44.46 ± 4.41	48.83 ± 14.34	50.90 ± 10.67	49.78 ± 16.81	0.858
AST (U/l)	28.94 ± 6.92	44.36 ± 24.80	46.42 ± 17.27	44.60 ± 46.09	31.44 ± 11.67	0.219
TB (μmol/l)	1.78 ± 1.00	3.82 ± 2.26	4.61 ± 2.97*	4.47 ± 3.13*	5.55 ± 3.82*	0.056
DBIL (μmol/l)	0.71 ± 0.54	1.78 ± 1.54*	1.73 ± 1.09*	1.53 ± 1.09	2.19 ± 1.18*	0.017
IDIL (μmol/l)	0.75 ± 0.61	2.17 ± 1.60*	2.88 ± 2.17^*^	2.96 ± 2.41^*^	3.23 ± 2.75*	0.048
TP (g/l)	64.73 ± 4.19	72.42 ± 5.84*	71.73 ± 4.81^*^	69.18 ± 6.44^*^	75.28 ± 3.74*	0.001
ALB (g/l)	32.87 ± 6.38	37.86 ± 4.13*	35.58 ± 4.29	33.78 ± 3.63	35.01 ± 4.54	0.127
GLB (g/l)	31.86 ± 5.16	34.56 ± 5.14	36.16 ± 6.83	35.40 ± 7.65	40.27 ± 8.19*	0.052
ALB/GLB	1.08 ± 0.34	1.13 ± 0.25	1.03 ± 0.27	1.02 ± 0.30	0.92 ± 0.29	0.613
γ-GT	79.69 ± 34.75	94.91 ± 28.80	77.25 ± 15.61	69.70 ± 12.68^#^	80.44 ± 22.19	0.254
LDH (U/l)	496.25 ± 114.07	618.64 ± 252.46	390.00 ± 54.63^#^	449.00 ± 107.65	389.38 ± 107.49^#^	0.021
ALP (U/l)	156.44 ± 67.95	146.46 ± 62.73	124.17 ± 56.59	138.90 ± 127.30	104.89 ± 84.16	0.595
Urea (μmol/l)	2.88 ± 0.85	4.50 ± 1.19*	4.98 ± 0.76*	5.82 ± 1.09*	5.70 ± 1.28*	0.001
Cr (μmol/l)	70.44 ± 22.00	66.00 ± 15.97	67.83 ± 20.24	51.00 ± 24.18	54.63 ± 17.37	0.259
CKMB (μg/l)	0.33 ± 0.22	1.04 ± 1.75	2.81 ± 2.30*	4.29 ± 3.14*	2.53 ± 2.24*	0.001
WBC (10^9^/l)	18.21 ± 5.45	14.29 ± 6.42	13.59 ± 5.41*	14.53 ± 4.61	11.99 ± 3.71*	0.057
RBC (10^12^/l)	6.76 ± 1.18	6.55 ± 1.24	6.97 ± 2.44	5.84 ± 0.65	6.28 ± 1.01	0.46
PLT (10^9^/l)	506.56 ± 123.72	409.75 ± 163.56	382.42 ± 154.30	386.00 ± 101.90	448.78 ± 208.50	0.191

*ADA* adenosine dehydrogenase,* ALB* albumin,* ALP* alkaline phosphatase,* ALT* alanine aminotransferase,* AST* aspartate aminotransferase,* CKMB* creatine kinase isoenzyme,* DBIL* direct bilirubin,* FBG* fasting blood glucose,* GLB* globulin,* HDL* high-density lipoprotein cholesterol,* IDIL* indirect bilirubin,* LDH* lactate dehydrogenase,* LDL* low-density lipoprotein cholesterol,* PLT* platelets,* RBC* red blood count,* TB* total bilirubin,* TC* total cholesterol,* TG* triglycerides,* TP* Total Protein,* WBC* white blood cell,* γ-GT* glutamyl transpeptidase

*P < 0.05 versus before modelling. ^#^*P* < 0.05 versus 2 months after modelling

### Dynamic changes in CMR parameters in pigs with the progression of diabetes

**Fig. 2:**
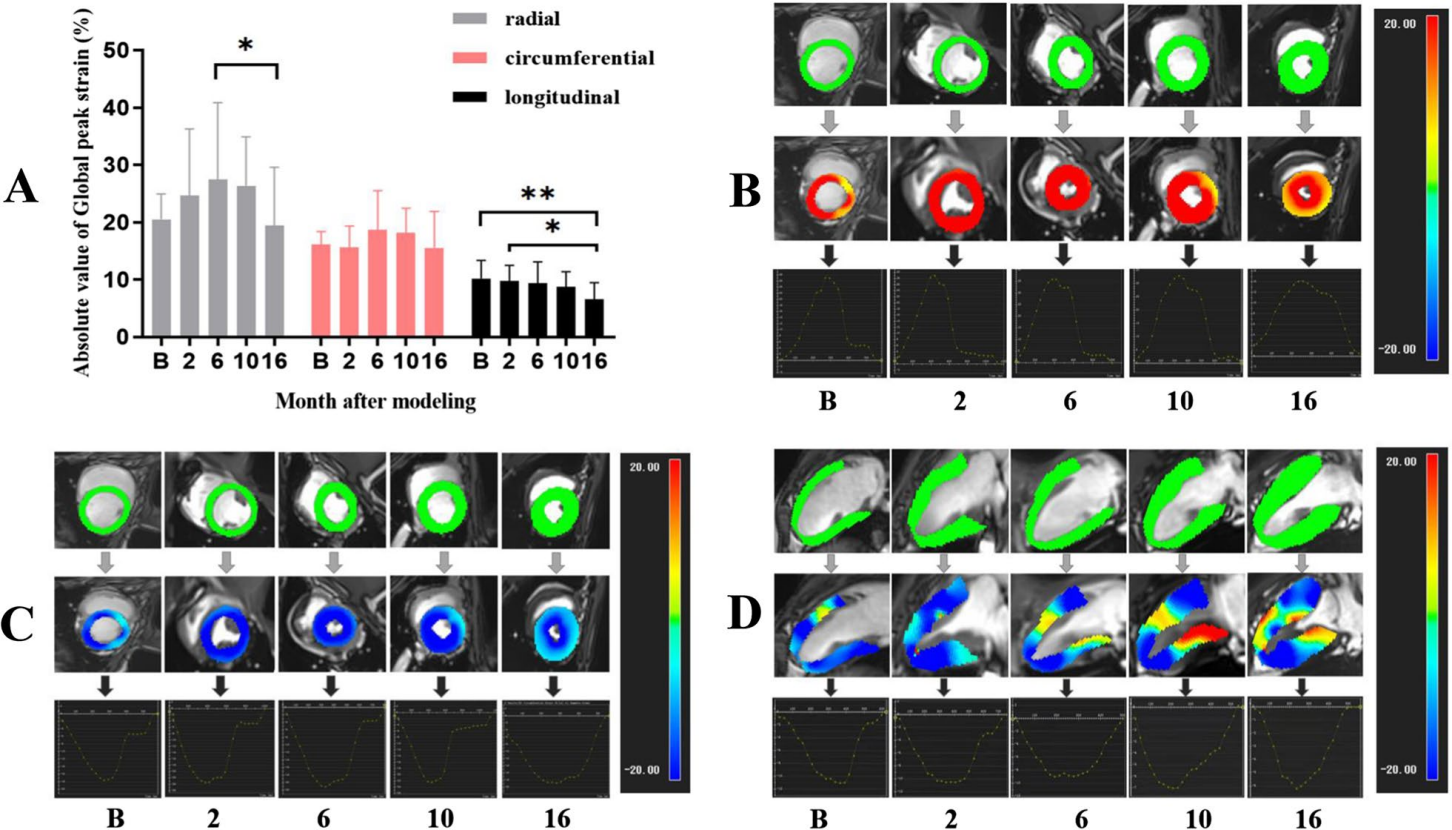
Comparison of global peak strain in three directions of the left ventricle in diabetic pigs at different scanning times. **A** The global peak strain of the left ventricle decreased gradually in the longitudinal direction, but increased first and then decreased in the radial direction. **B** Baseline. * *P* < 0.05; **P < 0.01. **B**–**D** LV radial, circumferential, longitudinal stain pseudocolour images of a diabetic pig at different timepoints. Top, end diastole; middle, end systolic; bottom, LV global peak strain curve. Baseline, GRPS = 27.05, GCPS= − 17.55, GLPS= − 11.07; 2 months after modelling, GRPS = 28.96, GCPS= − 21.03, GLPS = − 10.51; 6 months after modelling, GRPS = 35.81, GCPS = − 23.36, GLPS = − 8.94; 10 months after modelling, GRPS = 32.96, GCPS = − 22.51, GLPS = − 7.03; 16 months after modelling, GRPS = 16.95, GCPS = − 19.54, GLPS = − 6.19

The LV structure and function findings for the experimental pigs are shown in Table 2. Throughout the study period, there were no significant changes in ejection fraction, while the 80% diastolic volume recovery (DVR) increased significantly at 6 months after modelling (0.78 ± 0.08 vs. 0.67 ± 0.15), and the absolute value of GLPS (− 10.21 ± 3.15 vs. − 9.74 ± 2.78 vs. − 9.38 ± 3.71 vs. − 8.71 ± 2.68 vs. − 6.59 ± 2.90%) decreased gradually from the baseline data to 2, 6, 10, and 16 months after modelling. The GRPS increased in 6 months after modelling and then decreased 16 months after modelling. During the systolic period, the peak strain rate of the LV in all directions did not change significantly. During diastole, the longitudinal peak strain rate was lower than the original value 16 months after modelling (0.59 ± 0.25 vs. 1.09 ± 0.46), while the absolute value of radial peak strain rates at 16 months after modelling was lower than that at 6 months after modelling (− 1.69 ± 0.61 vs. -2.97 ± 1.24). The LV global peak strain changes in pigs at different timepoints are shown in Fig. 2A. The representative CMR strain manifestations of a pig at different timepoints are shown in Fig. 2B–D.

**Table 2:** Comparison of cardiac magnetic resonance results of LV structure and function in diabetic pigs at different scanning times

	Before modelling	2 months after modelling	6 months after modelling	10 months after modelling	16 months after modelling	*P* value
Geometric and functional						
EDV (ml)	32.43 ± 11.67	35.23 ± 10.12	42.08 ± 7.60*	33.45 ± 6.74	27.93 ± 11.49^&^	0.027
ESV (ml)	12.87 ± 7.08	14.88 ± 5.92	16.74 ± 11.01	10.33 ± 4.34&	7.96 ± 2.80^#&^	0.05
SV (ml)	19.56 ± 6.11	20.35 ± 7.05	25.34 ± 7.93	23.11 ± 4.91	19.97 ± 10.36	0.243
EF	60.30 ± 10.31	54.05 ± 10.14	56.50 ± 15.82	62.00 ± 9, 67	61.32 ± 13.63	0.250
HR	98.65 ± 20.57	92.85 ± 19.07	98.04 ± 20.91	96.15 ± 24.56	95.06 ± 24.84	0.963
CO	2.00 ± 0.67	1.92 ± 0.60	2.76 ± 1.14*#	2.44 ± 0.68	1.86 ± 0.91^&^	0.046
LVEDD	29.85 ± 4.47	31.30 ± 6.01	33.72 ± 3.68*	29.57 ± 5.20	25.81 ± 5.66^&^	0.014
LVESD	20.58 ± 3.84	21.18 ± 5.86	23.33 ± 6.60	19.52 ± 4.41	15.67 ± 3.47*^#&^	0.02
LV wall thickness	9.40 ± 1.94	9.77 ± 1.64	10.25 ± 2.01	10.51 ± 1.94	12.04 ± 1.43*^#^	0.018
MM (g)	40.15 ± 10.33	42.41 ± 13.09	51.81 ± 16.18*	52.66 ± 11.16*	54.28 ± 13.24*	0.036
LVRI (g/ml)	1.36 ± 0.45	1.26 ± 0.57	1.27 ± 0.36	1.52 ± 0.38	2.19 ± 0.97*^#&^	0.002
Time‒volume curve						
PER	104.14 ± 25.26	110.03 ± 34.56	128.90 ± 46.48	125.04 ± 28.20	104.97 ± 47.56	0.323
PFR	148.50 ± 55.87	136.74 ± 43.36	177.63 ± 79.13	167.18 ± 50.09	138.84 ± 65.46	0.407
PER/EDV	3.19 ± 0.82	2.93 ± 0.99	2.65 ± 0.95	2.99 ± 0.50	3.06 ± 0.60	0.521
PFR/EDV	4.46 ± 1.41	3.68 ± 1.41	3.54 ± 1.27	3.96 ± 0.80	3.97 ± 1.06	0.348
DVR80%	0.67 ± 0.15	0.71 ± 0.08	0.78 ± 0.08*	0.78 ± 0.07*	0.77 ± 0.06*	0.017
Strain						
GRPS (%)	20.55 ± 4.39	24.68 ± 11.61	27.48 ± 13.42	26.40 ± 8.55	19.45 ± 10.17^&^	0.239
GCPS (%)	− 16.14 ± 2.27	− 15.62 ± 3.73	− 18.67 ± 6.89	− 18.14 ± 4.37	− 15.56 ± 6.33	0.407
GLPS (%)	− 10.21 ± 3.15	− 9.74 ± 2.78	− 9.38 ± 3.71	− 8.71 ± 2.68	− 6.59 ± 2.90*^#^	0.084
GPSSR-R	1.56 ± 0.33	1.63 ± 0.53	1.79 ± 0.89	1.81 ± 0.68	1.54 ± 0.18	0.491
GPSSR-C	− 1.03 ± 0.19	− 0.94 ± 0.14	− 1.10 ± 0.25	− 1.09 ± 0.41	− 1.04 ± 0.08	0.587
GPSSR-L	− 0.88 ± 0.34	− 0.81 ± 0.19	− 0.71 ± 0.18	− 0.81 ± 0.30	− 0.71 ± 0.20	0.326
GPDSR-R	− 2.14 ± 0.60	− 1.97 ± 0.48	− 2.97 ± 1.24	− 2.74 ± 1.38	− 1.69 ± 0.61^&^	0.021
GPDSR-C	1.63 ± 0.32	1.55 ± 0.24	1.81 ± 0.94	1.66 ± 0.54	1.42 ± 0.41	0.601
GPDSR-L	1.09 ± 0.46	0.96 ± 0.35	0.87 ± 0.44	0.81 ± 0.32	0.59 ± 0.25*	0.082

*C* circumferential,* EDD* end-diastolic diameter,* EDV* end-diastolic volume,* ESD* end-systolic diameter,* EF* ejection fraction,* ESV* end-systolic volume,* GPS* global peak strain,* GPDSR* global peak diastolic strain rate,* GPSSR* global peak systolic strain rate,* L* longitudinal,* LV* left ventricular,* LVRI* left ventricular remodelling index,* PER* peak ejection rate,* PFR* peak filling rate,* R* radial;* SV* stroke volume

* *P* < 0.05 versus before modelling; ^#^*P* < 0.05 versus 2 months after modelling. ^&^*P* < 0.05 versus 6 months after modelling

LV mass increased gradually with the progression of the course of the disease (from the baseline data to 2, 6, 10 and 16 months after modelling, 40.15 ± 10.33 vs. 42.41 ± 13.09 vs. 51.81 ± 16.18 vs. 52.66 ± 11.16 vs. 54.28 ± 13.24 g). LV diameter increase first and then decrease after modelling. After 16 months of modelling, the LVRI of pigs increased compared with baseline data (16 months after modelling vs. baseline data, 2.19 ± 0.97 vs. 1.36 ± 0.45 g/ml).

### Correlations between LVRI and LV global peak strain in diabetic pigs

**Fig. 3:**
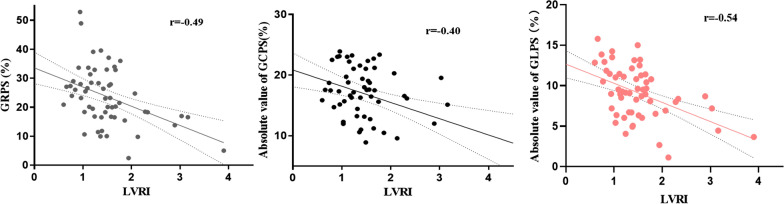
Correlations between LV global peak strain and LVRI in diabetic pigs.* LVRI* left ventricular remodelling index,* GRPS* radial global peak strain,* GCPS* circumferential global peak strain,* GLPS* longitudinal global peak strain. All *P* < 0.01

As shown in Fig. 3, significant linear correlations were observed between LV global peak strain and LVRI. There was a slight correlation between the absolute values of GRPS, GCPS and LVRI, (r = − 0.49 and − 0.40, respectively, *P* < 0.01). There was a moderate correlation between the absolute value of GLPS and LVRI, (r = − 0.54, *P* < 0.01).

### Histological analysis of myocardial ultrastructure and microstructure alterations in diabetic pigs

**Fig. 4:**
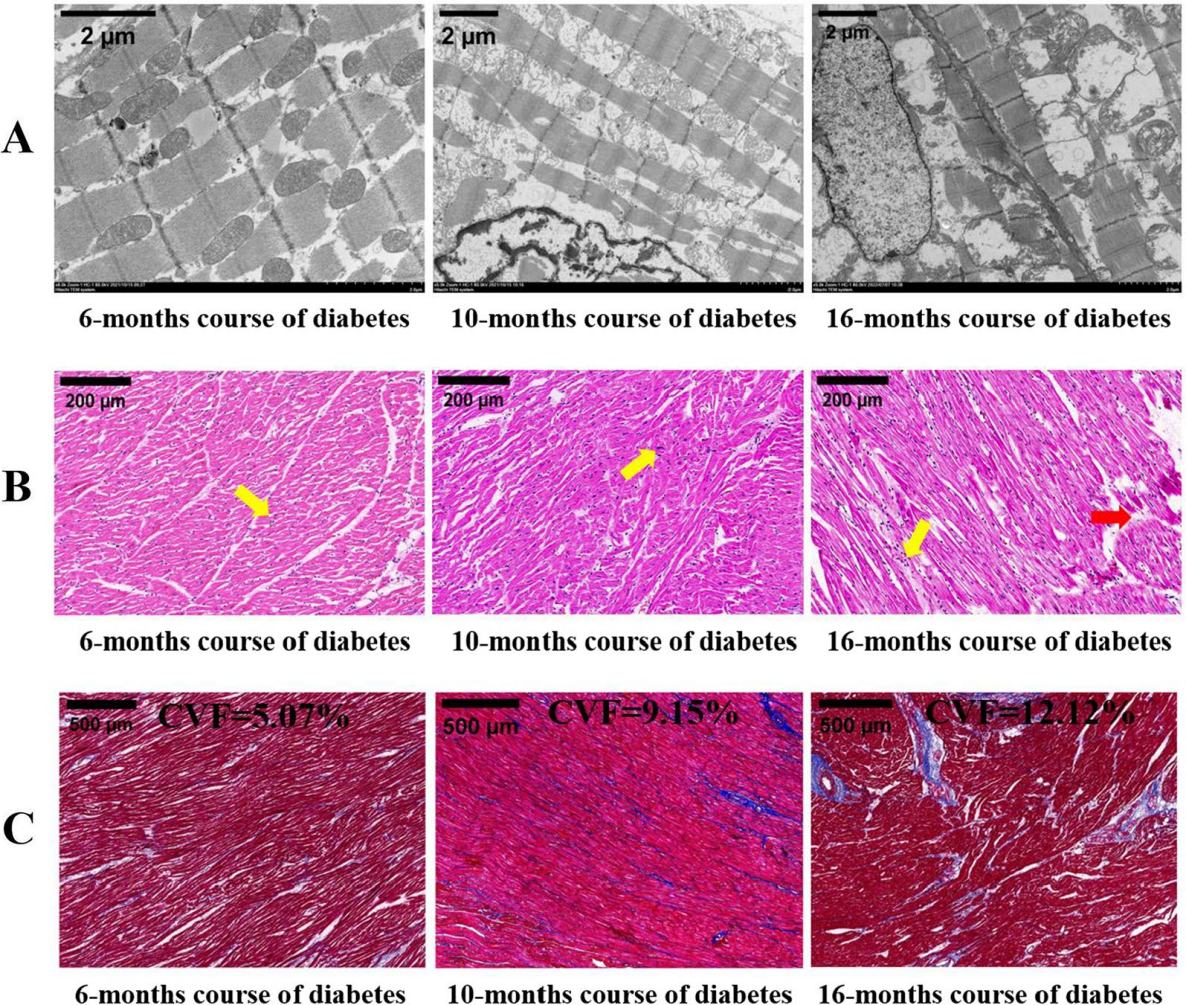
Transmission electron microscopy and pathological staining of the myocardium in diabetic pigs with different disease courses. ** A** Electron microscopy showed that with the progression of the disease, the mitochondrial damage of cardiomyocytes of diabetic pigs was aggravated, and myofilament injury appeared. **B** HE staining showed that the myocardial fibers were closely arranged, with occasional irregularities in nucleus size and arrangement (yellow arrow) at 6 months and 10 months. At 16 months, the local myocardial fibers were broken (red arrow) and the interfascicular interval of myocardium was widened.** C** Masson staining showed that myocardial interstitial fibrosis gradually aggravated with the progression of the disease.* CVF* collagen volume fraction

Using a transmission electron microscope **(**Fig. 4A**)**, it was observed that after six months of modelling, the myocardial mitochondria of the diabetic pigs were slightly swollen, and the myofilaments were arranged neatly and undamaged. Ten months after suffering from diabetes, mitochondrial swelling and partial vacuolation were observed. After sixteen months of diabetes, the mitochondrial damage of porcine cardiomyocytes was aggravated, and some myofilaments were atrophied and broken.

HE and Masson staining was used to observe the myocardial microstructure of pigs with different courses of diabetes **(**Fig. 4B, C**)**. HE staining showed irregularities in nucleus size and arrangement at six months and ten months after modelling. After sixteen months of diabetes, the local myocardial fibers were broken and the interval of myocardium was widened. Masson staining six months after modelling showed a small amount of collagen fibers around the blood vessels; Ten months after modelling, the myocardium atrophied locally, and fibrosis was obvious. After sixteen months of diabetes, Masson staining revealed obvious myocardial atrophy and diffuse interstitial fibrosis. With the prolongation of the course of diabetes, the proportion of myocardial fibrosis gradually increased.

### Reproducibility of CMR feature tracking and time‒volume curve parameters

As shown in Table 3, the intra- and interobserver agreements in the measurement of time ‒volume curve parameters (ICC = 0.866–0.955 and 0.863–0.923, respectively) and global LV peak strain (ICC = 0.925–0.987 and 0.887–0.958, respectively) were excellent.

**Table 3:** Inter- and intraobserver variability of the time‒volume curve and feature tracking

	Intraobserver (n = 15) ICC	95% CI	Interobserver (n = 15) ICC	95% CI
PER	0.912	0.759–0.969	0.867	0.633–0.943
PFR	0.955	0.873–0.986	0.902	0.765–0.934
PER/EDV	0.866	0.657–0.953	0.863	0.773–0.914
PFR/EDV	0.922	0.673–0.978	0.887	0.873–0.924
DVR80%	0.926	0.795–0.974	0.923	0.895–0.956
GRPS	0.925	0.878–0.963	0.887	0.705–0.960
GCPS	0.974	0.926–0.991	0.958	0.905–0.984
GLPS	0.987	0.966–0.996	0.937	0.883–0.975

*ICC* intraclass correlation coefficient,* PER* peak ejection rate,* PFR* peak filling rate,* EDV* end-diastolic volume,* GRPS* radial global peak strain,* GCPS* circumferential global peak strain,* GLPS* longitudinal global peak strain

All *P* < 0.001

## Discussion

The main findings of this study can be summarized as follows: (1) Intravenous injection of STZ could successfully induce the pig model of diabetic cardiomyopathy. (2) The primary manifestation of early cardiac dysfunction in diabetic pigs was the gradual decrease in LV diastolic function, followed by ventricular remodelling. (3) LV remodelling was related to myocardial strain, especially the change in longitudinal strain. (4) With the progression of the disease, the aggravation of cardiac dysfunction reflected by CMR was consistent with the degree of myocardial pathological damage in diabetic pigs.

STZ has selective toxicity to islet β cells. The animal model of diabetes induced by STZ is a widely used experimental method at present [15–17]. We successfully established 12 diabetes models in 16 Bama mini-pigs by a single high-dose STZ injection. The success rate of modelling in this experiment was similar to that of different breeds of pigs in other experiments, but hyperglycaemia was maintained for a longer time [16, 18]. The heart anatomy and the electrophysiological characteristics of pigs are more similar to those of humans than rodents, making pigs an ideal animal model for simulating human heart disease [19]. However, previous studies have lacked longitudinal studies on cardiac function and structure in diabetic pigs. Through continuous observation for 16 months, we concluded that STZ-induced diabetic pigs could be suitable subjects for the study of diabetic cardiomyopathy.

According to our results, the LV 80%DVR of diabetic pigs began to decrease in the early stage after modelling. It is suggested that the change in diastolic function may be the first manifestation of impaired LV function in diabetic pigs. In terms of myocardial strain, the longitudinal peak strain rate in the diastolic period after 16 months of modelling was lower than that before modelling, but the EF and systolic peak strain rate were still not significantly different from those recorded at baseline. These findings suggest that the time ‒volume curve and feature tracking technique can provide more sensitive imaging biomarkers than EF, which is consistent with past studies [7, 20, 21]. It is worth noting that in our experiment, except for the decrease in GLPS, GRPS and GCPS did not seem to decrease significantly in the early stage of diabetes in pigs, and even increased briefly at 6 months after modelling. This may be because the internal myocardium, which mainly causes the longitudinal movement of the heart, would bear the brunt of the damage caused by hyperglycaemia [22]. The slight increase in GRPS may also be the reason for the retention of EF in the early stage of diabetic cardiomyopathy.

In addition to the decline in cardiac diastolic function, myocardial hypertrophy and ventricular remodelling caused by diabetes are also considered to be closely related to the prognosis of the disease [23]. In our study, the LVRI of diabetic pigs did not change significantly within the first six months after modelling, but increased significantly in the later stage of follow-up. Besides, LVRI was negatively correlated with the absolute value of peak strain in three directions, and the correlation with GLPS was the strongest, suggesting that cardiac remodelling in the early stage of diabetes is closely related to the change in strain. Previously, Shao et al. found strain reduction and LV remodelling in three pigs with a 6-month course of diabetes, but they did not have a longer follow-up [18]. In previous clinical studies, compared to a BMI-matched control group, patients with diabetes showed LV concentric remodelling in the absence of hypertension [24]. This centripetal remodelling was considered to be related to cardiac steatosis and myocardial energy impairment in the subclinical stage [25–27]. We observed mitochondrial damage in myocardial cells in diabetic animals under an electron microscope, which well supported these conclusions.

The changes in LV function and structure caused by diabetic cardiomyopathy may take several years to show in clinical practice. In this study, through the cardiac pathological examination of diabetic pigs, cardiomyocytes were damaged in the early stage of diabetes, and further aggravated with the prolongation of the course of the disease. This shows that attention should be given to the early diagnosis of diabetic cardiomyopathy. In the past, studies on the pathogenesis of diabetic cardiomyopathy mainly came from basic research [28, 29]. It is believed that energy metabolism disorder, insulin resistance and angiotensin activity are the main factors directly affecting cardiomyocytes caused by elevated blood glucose [30–32]. The progress of technology, especially the rapid development of imaging methods, provides a powerful method for the evaluation of early pathological transformation in the diabetic heart. At present, many drugs have been proven to effectively improve the oxidative stress and fibrosis of rodent cardiomyocytes caused by diabetes [33–36]. It is believed that, with the continuous elucidation of the pathological mechanism, many drugs will be further verified in animal models more similar to the human body, and will gradually be used in clinical practice.

There are some limitations in this study. First, considering the maintenance of the model, we administered insulin intervention to some pigs with hyperglycaemia during follow-up, which may make our results somewhat different from the changes in cardiac structure and function under sufficient hyperglycaemia exposure. In addition, only three animals were selected for pathological examination to preserve as many samples as possible for follow-up, thus our findings with the cardiac microstructure in diabetic pigs need to be validated by more biopsy results. Finally, considering the feasibility of the experiment, we adopted a self-control experimental design, and did not take gender as a variable. The possible mixed effects of gender need to be analyzed in future studies.

## Conclusion

In this study, the early changes in LV function and structure in STZ-induced diabetic Bama mini-pigs were observed by CMR time ‒volume curve and feature tracking techniques, which were mainly characterized by a decrease in LV diastolic function and myocardial stress changes with ventricular remodelling. The change in the quantitative parameters of CMR was consistent with the corresponding degree of myocardial microstructure damage. Implementing CMR biomarkers to evaluate the cardiac characteristics of diabetic pigs in different stages may become an important link between basic research and clinical treatment in the future.

## Supplementary Information

**Additional file 1: Fig. S1**. CMR myocardial tracking in short-axis and long-axis cine images of a porcine heart at end-diastole and end-systole.The LV epicardial boundary (green) and LV endocardial boundary (red) were drawn manually, and the software automatically tracked myocardial motion and calculated LV strain parameters.** Fig. S2**. Changes in blood glucose in pigs after first STZ injection.Blood glucose fluctuated irregularly in the first 24 h after ST injection. After 36 h, the blood glucose of the successfully modelled pigs stabilized at a high level.

## Data Availability

The datasets used and/or analyzed during the current study are available from the corresponding author on reasonable request.

## Abbreviations

CMR: Cardiac magnetic resonance
EDD: End-diastolic diameter
EDV: End-diastolic volume
EF: Ejection fraction
ESD: End systolic diameter
ESV: End-systolic volume
FBG: Fasting blood glucose
GCPS: Global circumferential peak strain
GLPS: Global longitudinal peak strain
GRPS: Global radial peak strain
HDL: High-density lipoprotein cholesterol
LDL: Low-density lipoprotein cholesterol
LV: Left ventricular
LVRI: Left ventricular remodelling index
MRI: Magnetic resonance imaging
PER: Peak ejection rate
PFR: Peak filling rate
STZ: Streptozotocin
SV: Stroke volume
