# Supplementary material for: Early longitudinal changes in left ventricular function and morphology in diabetic pigs: evaluation by 3.0T magnetic resonance imaging
Source: Cardiovasc Diabetol. 2023 Jan 10;22:6. doi: 10.1186/s12933-022-01734-y (PMC9830732; doi:10.1186/s12933-022-01734-y)
Supplement: Supplementary file 1 — Additional file 1: Fig. S1. CMR myocardial tracking in short-axis and long-axis cine images of a porcine heart at end-diastole and end-systole.The LV epicardial boundary (green) and LV endocardial boundary (red) were drawn manually, and the software automatically tracked myocardial motion and calculated LV strain parameters. Fig. S2. Changes in blood glucose in pigs after first STZ injection.Blood glucose fluctuated irregularly in the first 24 h after ST injection. After 36 h, the blood glucose of the successfully modelled pigs stabilized at a high level. [file 12933_2022_1734_MOESM1_ESM.docx]

**
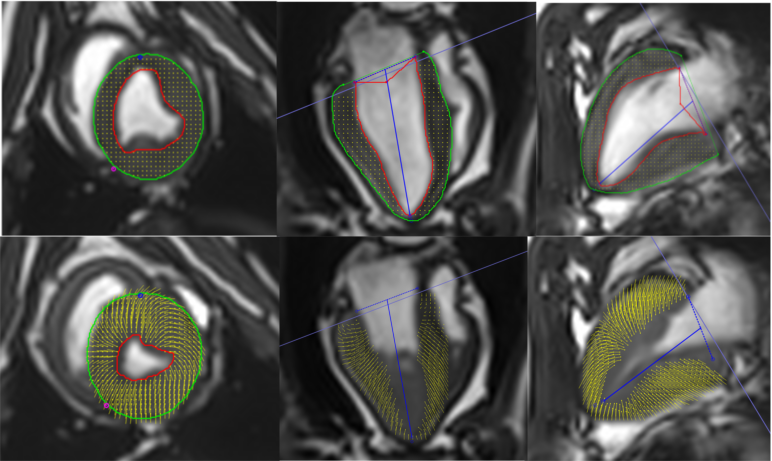
**

**Additional file 1: Figure S1. CMR myocardial tracking in short-axis and long-axis cine images of a porcine heart at end-diastole and end-systole.**

**The LV epicardial boundary (green) and LV endocardial boundary (red) were drawn manually, and the software automatically tracked myocardial motion and calculated LV strain parameters.**

**
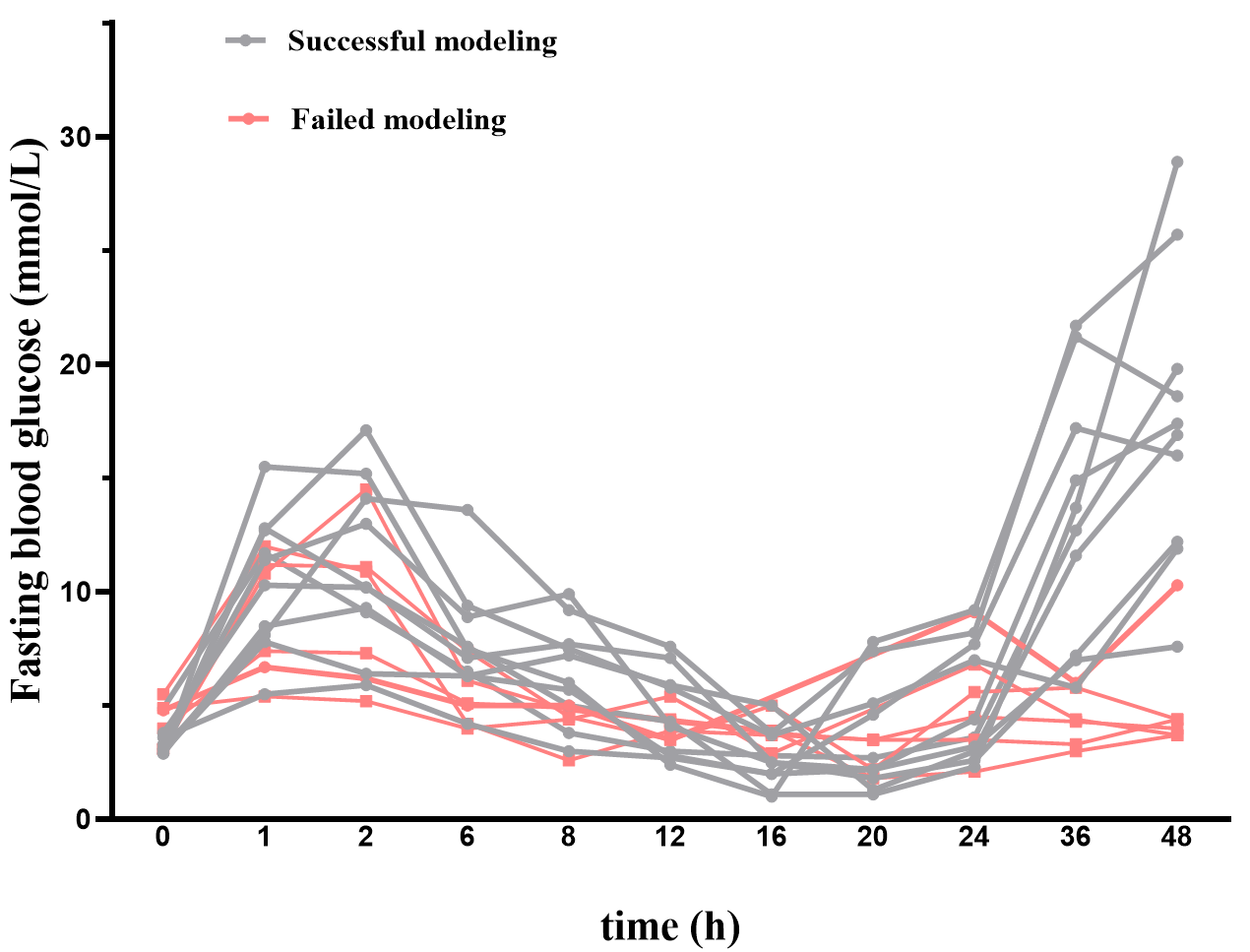
**

**Additional file 1: Figure S2. Changes in blood glucose in pigs after first STZ injection.**

**Blood glucose fluctuated irregularly in the first 24 hours after ST injection. After 36 hours, the blood glucose of the successfully modelled pigs stabilized at a high level.**
